# First Barnacle (*Amphibalanus amphitrite*) Adhesion Strength Data on the Self-Polishing Coatings Off the Aegean Sea

**DOI:** 10.1021/acsomega.3c03948

**Published:** 2023-09-08

**Authors:** Ibrahim Kirkiz, Levent Cavas

**Affiliations:** † Graduate School of Natural and Applied Sciences, Department of Biotechnology, Dokuz Eylül University, Kaynaklar Campus, İzmir 35390, Türkiye; ‡ Faculty of Science, Department of Chemistry (Biochemistry Division), Dokuz Eylül University, Kaynaklar Campus, İzmir 35390, Türkiye

## Abstract

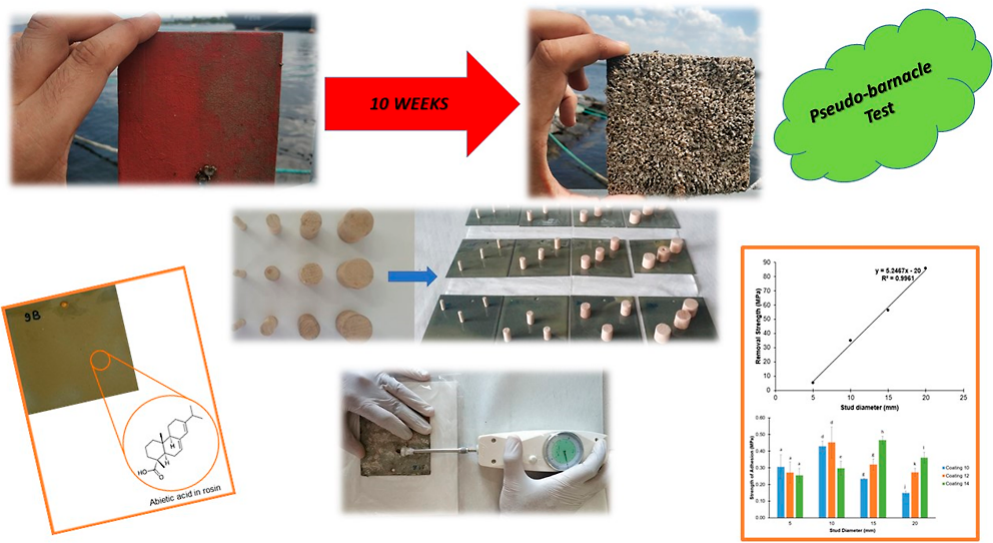

The aim of this work is to study the adhesion strength of *Amphibalanus amphitrite* in the İzmir Bay and compare the results with the pseudobarnacle adhesion test. Normally, adhesion tests are performed to evaluate the performance of the antifouling coatings, but the test results can also be used to predict biofouling cleaning process efficacy. The biofouling process is highly dependent on environmental conditions. For this reason, laboratory tests are required to perform the performance tests on self-polishing coatings in cases where living organisms cannot be reached. For this purpose, different self-polishing antifouling coatings have been formulated. Field tests for the coatings were carried out in the Aegean Sea for 10 weeks. After 10 weeks, barnacle and pseudobarnacle adhesion tests were conducted on coatings. When the results were compared, similarity was observed between the adhesion strength of barnacles and pseudobarnacles with 10 mm diameter on coating with the rosin/xylene/BaSO_4_ (40:40:20 w/w %). The adhesion strength of barnacles and pseudobarnacles on the coating 12 was found to be 0.46 and 0.45 MPa, respectively. In conclusion, the present study exhibits the first data related to the adhesion strength of *A. amphitrite* on rosin-based self-polishing coatings in the Aegean Sea. Moreover, based on field tests, a pseudobarnacle adhesion test methodology was developed to mimic barnacles and the correlation between barnacle and pseudobarnacle tests was examined.

## 1 Introduction

Biofouling is the unwanted accumulation of micro- and macro-organisms onto the surfaces that they have contact with.^1,2^ This accumulation of organisms causes serious problems in industries such as filtration systems, powerplants, aquaculture cages, and ship transportation.^3−5^ Biofouling of marine organisms on to surfaces that are immersed in seawater is called marine biofouling.^6,7^ Antifouling refers to any efforts for preventing the accumulation of marine organisms on the surfaces.^8^ Antifouling technologies are used to combat biofouling in marine industries. In particular, biofouling on ships’ hulls has negative effects on ship industries. The accumulation of these organisms on the smooth surface of the ship increases the friction force, causing the ship’s speed and maneuverability to decrease, which causes more fuel consumption and results in increased carbon emission.^9,10^ Biofouling on ships’ parts also causes transport of new species into other environments.^11^ With this issue, biodiversity of marine ecosystems will differ. To eliminate these issues, dry-docking operations are used more often, but these operations require time, resources, and labor. Biofouling also causes series problems to fish farms. Formation of biofouling on aquaculture fish nets blocks most of the mesh openings, thus preventing material and water exchange.^12^

Among the antifouling technologies, tributyltin (TBT) self-polishing paints were the most effective. It was even thought that these coatings were a permanent solution to biofouling problem.^13^ However, when the negative effects of these coatings on living organisms are understood, the use of TBT coatings were prohibited by the International Maritime Organization.^14^ After this ban, serious regulations and inspections were brought to the use of antifouling paints.^15^ When this is the case, the importance of biocide-free fouling release and self-polishing coatings has increased. The general principle of fouling release coatings is based on the removal of biofouling organisms on the surface because of dynamic movements in the sea, by reducing the adhesion strength between the surface and organisms.^16,17^ On the other hand, self-polishing coatings release their contents, usually biocides, continuously and consistently due to the binder being soluble in seawater.^18^ Field tests are required to gain information on the performance of these biocide-free coatings. These field tests are usually performed according to ASTM D3623—78a,^19^ which is used to determine the rate of biofouling of the surface. Although this method is suitable for coatings containing biocides, it is not sufficient for biocide-free fouling release coatings.^20^ Since biocide-free fouling release coatings rely on adhesion strength, new methods were developed for the measurements of adhesion strength. One of these methods is to measure barnacle adhesion strength by shear force. Barnacles were chosen as test subjects because they can adhere to most surfaces, and they are found in most seas as biofouling organism.^21^ The biggest problem with this method is that the barnacle population is not the same in all seasons. Especially in summer, the number of fouling organisms increases, while in winter, it decreases. For this reason, a laboratory test, the pseudobarnacle adhesion test, which can be similar to the barnacle adhesion test, has been developed.^22^

Rosin and its derivatives are commonly used materials for self-polishing coatings, but they are not preferred as fouling release coatings. In this study, barnacle and pseudobarnacle adhesion tests on rosin-based self-polishing coatings were investigated for the first time in the Aegean Sea. In this way, how rosin-based coatings without biocide would perform in antifouling performance test were investigated. Thus, the correlation between barnacle and pseudobarnacle tests in biofouling species of the Aegean Sea was examined in this study. In addition, *t*-test and correlation tests were performed to find out which variable was significant and how much it affected the result.

## 2 Materials and Methods

In order to prepare coatings, technical grade rosin, zinc rosinate, xylene, CaCO_3_, ZnO, and BaSO_4_ (Tekkim) were used. The PVB used in the coatings was provided by Shark Solutions. Metal plates (10 × 10 cm^2^) were provided by Dokuz Eylul University Technical Workshops. The diameters of the barnacles were measured by using a Vernier caliper (Leo, 0–150 × 0.05 mm). To measure the removal strength of the barnacles, an analog dynamometer (Loyka NK-200) was used.

### 2.1 Preparation of Biocide-Free Self-Polishing Coatings

To prepare rosin and zinc rosinate-based coatings, rosin or zinc rosinate was dissolved in xylene at 60 °C and at 200 rpm using a magnetic stirrer. After obtaining a homogenous texture, supportive materials like CaCO_3_, ZnO, and BaSO_4_ were added to the mixture. The final mixture was stirred until homogenous and desired texture obtained. Polyvinyl butyral (PVB)-based coatings with different percentages were also used in the field tests. There were a total of 23 different formulations that were evaluated with the field tests, and their contents are given in Table S1.

After preparing all the coatings, a series of 10 × 10 cm^2^ metal plates were prepared for the field tests. Holes with a diameter of 5 mm were drilled in the middle of one side of all plates. Afterward, the plates were sanded, and their surfaces were cleaned with methanol. After these processes, the plates were completely painted with an anticorrosive primer to prevent from rusting in seawater. Then, the prepared coatings were applied on the panels, and they were left in the open area until completely dry. After 2 weeks, they were fixed to polyvinyl chloride (PVC) pipes to be hung in the seawater.

### 2.2 Field Tests

The metal plates painted with different coatings were immersed in seawater at a depth of 50 cm in İnciraltı coast between July and September (38°24′48.7″N, 27°02′03.9″E, İzmir, Türkiye). After 10 weeks, plates were removed from seawater and brought to the laboratory to conduct adhesion tests. To evaluate field test results, the scoring system is used. For this scoring system, a system previously available in the literature was used.^23,24^ The scoring system is as follows:

Level I: there are no fouling organisms on the surface.

Level II: there is thin microfilm layer formation on the surface.

Level III: there is a thick microfilm layer and macrofoulant spores on the surface.

Level IV: there are soft and hard foulers on the surface (<50% overall coverage).

Level V: the surface is heavily fouled (>50% overall coverage).

### 2.3 Barnacle Adhesion Tests

According to ASTM D 5618—94,^25^ the test should be carried out on barnacles with a diameter of 5–20 mm, approximately 20 mm from the edges of the plates and not in contact with other organisms. Therefore, after bringing panels into laboratory, their surfaces must be cleaned from other biofouling organisms such as *Ulva lactuca* and *Hydroides elegans*. *H. elegans* tubes were carefully scraped from the surface to perform the test properly. After cleaning and isolating the barnacles, their diameters were measured using a caliper. Since barnacles are not in a perfect circular shape, four diameters were measured in four different positions (0, 45, 90, and 135°). Then, the average base diameters (*d*_a_) of the barnacles were calculated. After obtaining average base diameters, the base area for the barnacles can be calculated using the following formulation
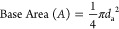


For measuring the removal strengths of barnacles, an analog dynamometer (Loyka NK 200) was used. An 8 mm rod and a suitable probe were attached to the dynamometer before the tests. For the shear force measurements, plates and the dynamometer were placed parallelly, and the force was applied to the barnacles until they were removed from the surface. While performing the tests, care was taken not to disturb the integrity of the barnacles. The barnacles that were crushed during the test were not included in the results. When taking note of the results, the results in which the barnacles were completely detached from the surface were considered.

For the calculation of the strength of adhesion, the measured removal strength of the barnacle (Newtons, *F*) is divided by the base area of the barnacle (millimeter square, *A*)
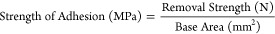


### 2.4 Pseudobarnacle Adhesion Tests

The same procedure was followed in pseudobarnacle adhesion test. Instead of using live barnacles, wooden pseudobarnacles with known diameters were adhered to the surfaces with epoxy (Pattex Power Epoxy). These pseudobarnacles can usually be aluminum studs, or made of wood. The same rules and calculations for the barnacle adhesion tests were also applied to this test. One of the important points to be considered is that excess adhesive should be cleaned after the pseudobarnacles are adhered to the surfaces.

**Figure 1:**
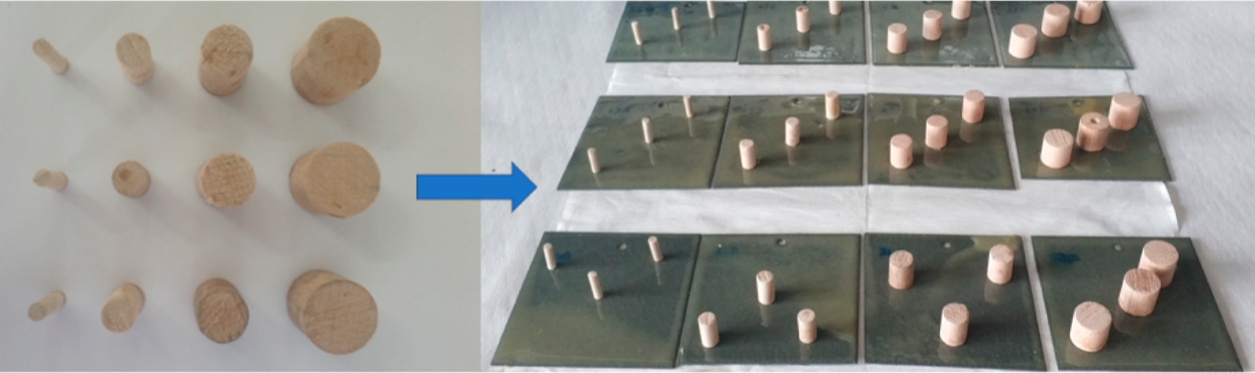
Wooden pseudobarnacles that were used in the adhesion tests.

In the literature, aluminum studs with fixed-known diameters (mostly 7 or 10 mm) are used as pseudobarnacles.^26−29^ However, barnacles grow by adding calcium carbonate to their shell plates, which is called calcification.^30^ Thus, they are found in different sizes in seawater. It was also found that the barnacle adhesion strength was affected by the size and age of the barnacles.^21^ Therefore, pseudobarnacles of different sizes were used to ensure accurate comparison with live barnacles in this study. Pseudobarnacle tests were repeated 3 times for each coating and stud diameter. The view of the pseudobarnacles is shown in Figure 1.

### 2.5 Statistical Test

Student’s *t* test and correlation test in Excel (Microsoft Office) were used to compare data. Statistical significance was set at 0.05.

## 3 Results and Discussion

### 3.1 Field Test Results

The biofouling progression on the coated panels is given in Figures S1–S10. The plates were immersed in İnciraltı/İzmir (Türkiye) between July and September 2022. When the panels were examined after test period is over, it was observed that *H. elegans*, *U. lactuca,* and *Bugula neritina* species were dominant on the surfaces. At first glance, no *Amphibalanus amphitrite* was observed on the surfaces. However, the presence of *A. amphitrite* was revealed when *H. elegans* tubes were carefully scraped from the surfaces. Based on this, it was concluded that *A. amphitrite* cyprids settle on surfaces early, while other species foul on to them.

The biofouling development on the surfaces of the plates kept in sea water for 10 weeks can be observed in Figures S1–S10. On the other hand, performance scores based on figures are given in Table 1.

**Table 1:** Antifouling Performance Scores of the Prepared Coatings

coatings	coating type	antifouling performance score			
		after 2 weeks	after 4 weeks	after 7 weeks	after 10 weeks
uncoated	-	I	IV	V	V
anticorrosive primer coated	primer	I	V	V	V
commercial antifouling paint	commercial	-	II	III	-
1	rosin	II	V	V	V
2	rosin	II	IV	V	V
3	rosin	-	V	V	-
4	zinc rosinate	-	III	V	-
5	zinc rosinate	-	III	IV	-
6	zinc rosinate	-	III	IV	-
7	PVB	III	V	V	V
8	PVB	III	V	V	V
9	PVB	III	IV	V	V
10	rosin	II	III	V	V
11	rosin	II	III	V	V
12	rosin	II	IV	V	V
13	rosin	II	III	V	V
14	rosin	II	IV	V	V
15	rosin	II	III	V	V
16	zinc rosinate	III	IV	V	V
17	zinc rosinate	III	IV	V	V
18	zinc rosinate	III	IV	V	V
19	zinc rosinate	III	III	V	V
20	zinc rosinate	III	IV	V	V
21	zinc rosinate	III	IV	IV	V
22	rosin	II	IV	V	V
23	zinc rosinate	-	II	IV	-

### 3.2 Barnacle Adhesion Test Results

**Figure 2:**
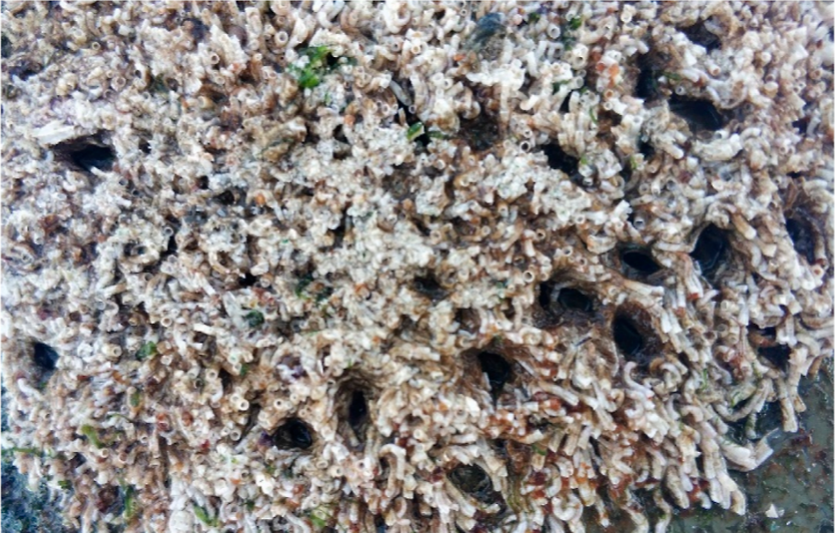
*A. amphitrite* living under *H. elegans*.

Although 23 different coatings were prepared for the field test, barnacle adhesion tests could be performed on only 4 coatings (coating 4, 10, 12, and 14) because barnacles did not attach to all surfaces. Even though most of the surfaces had a level V antifouling score after 10 weeks, there were very few barnacles on the surfaces at first glance. There was a density of *H. elegans* on the surfaces and some barnacles were visible from the cavities left by *H. elegans* (Figure 2). This being the case, the *H. elegans* tubes were carefully scraped. After scraping, the barnacles became visible.

However, the tests could not be carried out. The barnacles, whose surface was densely covered, died because they could not feed. What was revealed after scraping were the hollow outer shells of the barnacles. Some of the remaining coatings had living barnacles. However, the tests could not be performed again because the barnacles were in contact with each other, causing the measured removal strength to be inaccurate. Considering the above mentioned problems and ASTM D 5618—94, 4 coatings (coating 4, 10, 12, and 14) were found to be suitable for performing the adhesion test without any problems.

Table 2 shows barnacle adhesion strengths on coating 4 [25% (w/w) zinc rosinate, 75% (w/w) xylene], and a high correlation coefficient (*r* = 0.86) was found between the diameter and removal strength of barnacles.

**Table 2:** Adhesion Strengths of *A. amphitrite* on the Coating 4

diameter (mm)	removal strength (N)	base area (mm^2^)	strength of adhesion (MPa)
8	12	44	0.27
9	17	57	0.30
9	17	64	0.27
11	20	95	0.21
11	20	95	0.21
11	27	95	0.28
11	23	95	0.24
12	25	104	0.24
12	32	113	0.28
12	35	113	0.31
12	38	113	0.34
13	38	133	0.29
13	29	133	0.22

Table 3 shows the adhesion strengths of *A. amphitrite* on the coating 10 [40% (w/w) rosin, 40% (w/w) xylene, 20% (w/w) CaCO_3_]. The correlation coefficient (*r* = 0.87) between diameter and removal strength was high. In fact, this correlation coefficient is the highest value calculated among coatings 4, 12, and 14.

**Table 3:** Adhesion Strengths of *A. amphitrite* on the Coating 10

diameter (mm)	removal strength (N)	base area (mm^2^)	strength of adhesion (MPa)
4	12	13	0.96
5	10	20	0.51
6	11	28	0.39
7	21	38	0.55
7	15	38	0.39
7	24	38	0.62
8	16	50	0.32
8	14	50	0.28
8	15	50	0.30
9	22	64	0.35
9	21	64	0.33
10	34	79	0.43
15	40	177	0.23

On coating 12 [40% (w/w) rosin, 40% (w/w) xylene, 20% (w/w) BaSO_4_], there were not many *A. amphitrite*, but they were well positioned for the adhesion test. According to Table 4, a high correlation coefficient (*r* = 0.86) was found between diameter and removal strength.

**Table 4:** Adhesion Strengths of *A. amphitrite* on the Coating 12

diameter (mm)	removal strength (N)	base area (mm^2^)	strength of adhesion (MPa)
7	25	38	0.65
7	11	38	0.29
10	36	79	0.46
12	38	113	0.34

Using the data in Table 5, a high correlation coefficient (*r* = 0.81) was found between diameter and removal strength on coating 14 [40% (w/w) rosin, 40% (w/w) xylene, 10% (w/w) CaCO_3,_ 10% (w/w) BaSO_4_].

**Table 5:** Adhesion Strengths of *A. amphitrite* on the Coating 14

diameter (mm)	removal strength (N)	base area (mm^2^)	strength of adhesion (MPa)
3	9	7	1.27
4	7	13	0.56
5	12	20	0.61
7	24	38	0.62
7	16	38	0.42
9	27	64	0.42
9	11	64	0.17
11	30	95	0.32
11	18	95	0.19
12	31	113	0.27
12	33	113	0.29

### 3.3 Pseudobarnacle Adhesion Test Results

Wooden pseudobarnacles with 5, 10, 15, and 20 mm diameters were prepared to be used in the experiments. These pseudobarnacles were glued to surfaces with Pattex Power Epoxy, a fast-drying epoxy. The tests were carried out with samples that were dried for 1 and 6 h. To compare both adhesion tests, the coatings 10, 12, and 14 were chosen for pseudobarnacle adhesion tests. Since all 3 coatings are rosin-based, the effects of formulations on adhesion strength were also minimized. At the same time, the same tests were also applied to the plates coated with anticorrosive primer to determine if the coating affects the adhesion strength. Table 6 shows the results of pseudobarnacle tests on anticorrosive primer coated panels after 1 and 6 h of drying. The studs with a diameter of 20 mm could not be detached from the surface after both drying times. Since the dynamometer used can measure up to 200 N, the results were given as greater than 200 N. The strengths of adhesions were not calculated because the removal force could not be exactly known. Apart from that, the removal strengths of all studs were higher compared to the coatings 10, 12, and 14, except for the 5 mm diameter studs.

**Table 6:** Pseudobarnacle Test Results on Anticorrosive Primer Coated Panels

	diameter (mm)	mean removal strength (N)	base area (mm^2^)	strength of adhesion (MPa)
1 h of drying	5	10	20	0.51
	10	37	79	0.47
	15	153	177	0.87
	20	>200	314	N/A
6 h of drying	5	17	20	0.84
	10	96	79	1.22
	15	159	177	0.90
	20	>200	314	N/A

After conducting pseudobarnacle tests on the coatings 10, 12, and 14, data in Table 7 were obtained. These results were obtained after drying epoxy for 6 h. According to Table 7, high correlation coefficients were found between diameter and removal strength on the coatings 10, 12, and 14 (*r* = 0.96, 0.94, and 0.96, respectively).

**Table 7:** Pseudobarnacle Test Results on All Coatings after 6 h of Drying (±Standard Deviation)

	diameter (mm)	mean removal strength (N)	base area (mm^2^)	strength of adhesion (MPa)
coating 10	5	13	20	0.66 ± 0.291
	10	79	79	1.01 ± 0.113
	15	83	177	0.47 ± 0.088
	20	157	314	0.50 ± 0.043
coating 12	5	18	20	0.90 ± 0.064
	10	42	79	0.54 ± 0.174
	15	64	177	0.36 ± 0.030
	20	149	314	0.47 ± 0.094
coating 14	5	19	20	0.97 ± 0.181
	10	84	79	1.07 ± 0.178
	15	160	177	0.90 ± 0.142
	20	165	314	0.52 ± 0.054

**Figure 3:**
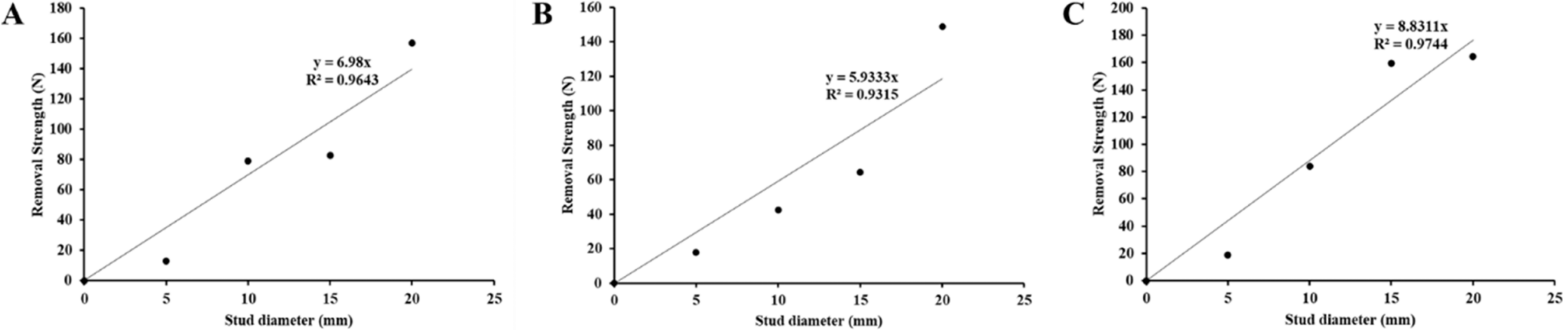
Relationship between removal strength and stud diameter on (A) coating 10, (B) coating 12, and (C) coating 14 after drying 6 h.

Figure 3 shows the relationship between removal strength and wooden stud diameter on all three coatings. As can be seen from Figure 1, all studs had three replicates on each of the coatings, because the mean removal strengths were used in the graphs. Pseudobarnacle adhesion tests on all coatings gave acceptable *R*^2^ values (*R*_10_^2^ = 0.9643, *R*_12_^2^ = 0.9315, and *R*_14_^2^ = 0.9744).

**Figure 4:**
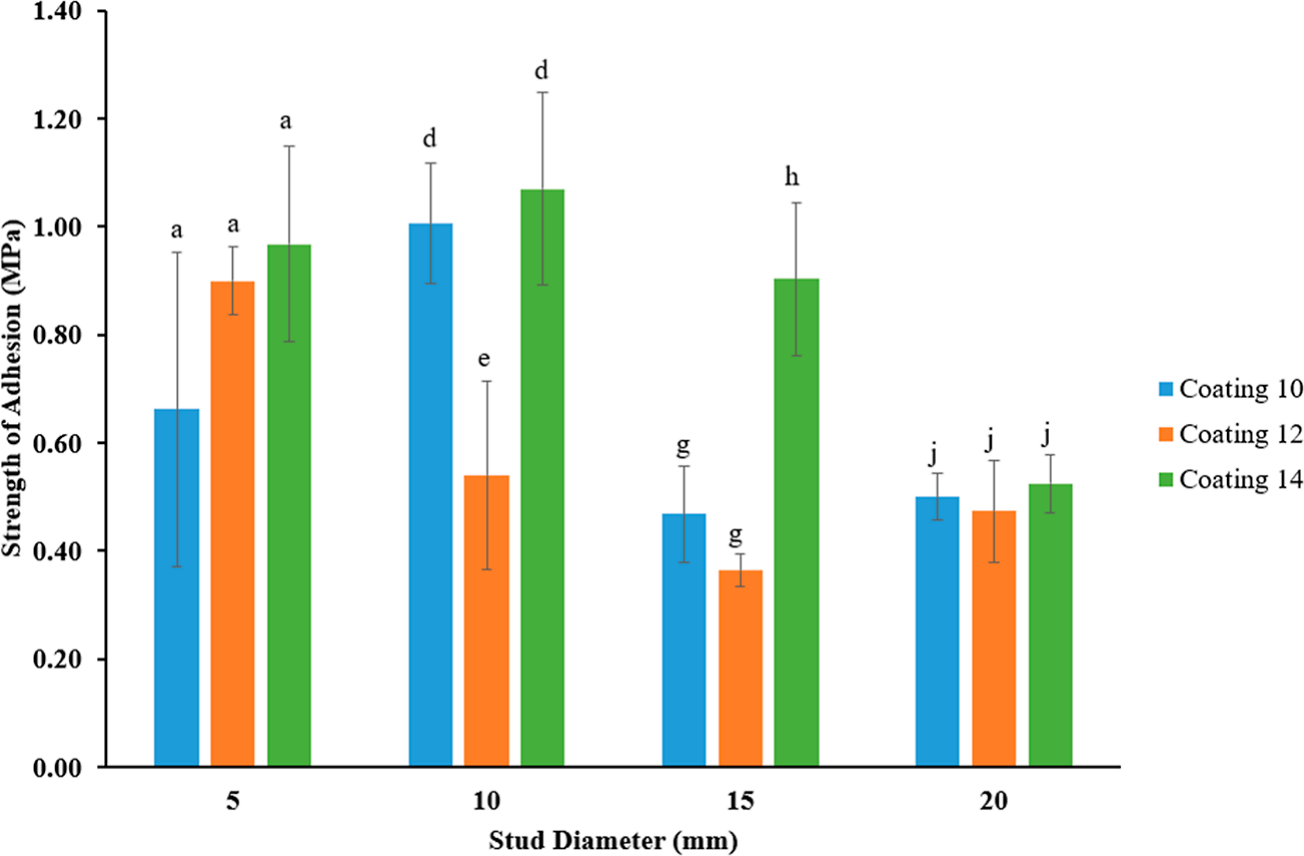
Adhesion strengths of different-sized pseudobarnacles on different coatings after 6 h drying.

Figure 4 shows the comparison of the strength of adhesion on all three coatings. Although the removal strength increases with the increasing stud diameter, the strength of adhesion differs in each coating and diameter. There is no statistical difference (*p* > 0.05) in the strengths of the adhesion of 5 mm studs among the coatings 10, 12, and 14. For the 10 mm studs, although there is a significant difference (*p* < 0.05) between the strength of adhesions of the coatings 10 and 12, we could not observe a significant difference (*p* > 0.05) between the coatings 10 and 14. Adhesion strengths of 15 mm studs had no significant difference (*p* > 0.05) on the coatings 10 and 12, but there is a difference (*p* < 0.05) on the coating 14. Lastly, for the 20 mm studs, there is no statistical difference (*p* > 0.05) between all 3 coatings.

After conducting pseudobarnacle tests, the data in Table 8 were obtained. Even though studs with 5 mm diameter were weakly adhered to the surfaces, correlation coefficient for 3 coatings were high (*r*_10_ = 0.93, *r*_12_ = 0.99, *r*_14_ = 0.98).

**Table 8:** Pseudobarnacle Test Results on All Coatings after an Hour of Drying (±Standard Deviation)

	diameter (mm)	mean removal strength (N)	base area (mm^2^)	strength of adhesion (MPa)
coating 10	5	6	20	0.31 ± 0.072
	10	34	79	0.43 ± 0.032
	15	42	177	0.23 ± 0.003
	20	47	314	0.15 ± 0.011
coating 12	5	5	20	0.27 ± 0.064
	10	35	79	0.45 ± 0.031
	15	56	177	0.32 ± 0.033
	20	86	314	0.27 ± 0.024
coating 14	5	5	20	0.25 ± 0.042
	10	23	79	0.30 ± 0.042
	15	82	177	0.47 ± 0.023
	20	113	314	0.36 ± 0.033

**Figure 5:**
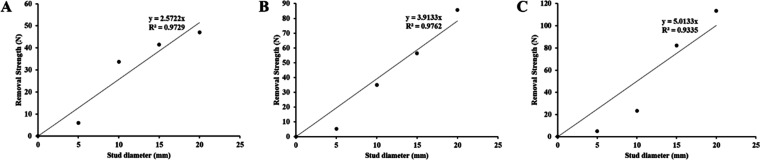
Relationship between removal strength and stud diameter on (A) coating 10, (B) coating 12, and (C) coating 14 after drying an hour.

Figure 5 shows the relationships between removal strength and stud diameter that dried for an hour. As can be seen from the graphs, all 3 coatings have high *R*^2^ values (*R*_10_^2^ = 0.9729, *R*_12_^2^ = 0.9762, and *R*_14_^2^ = 0.9335).

**Figure 6:**
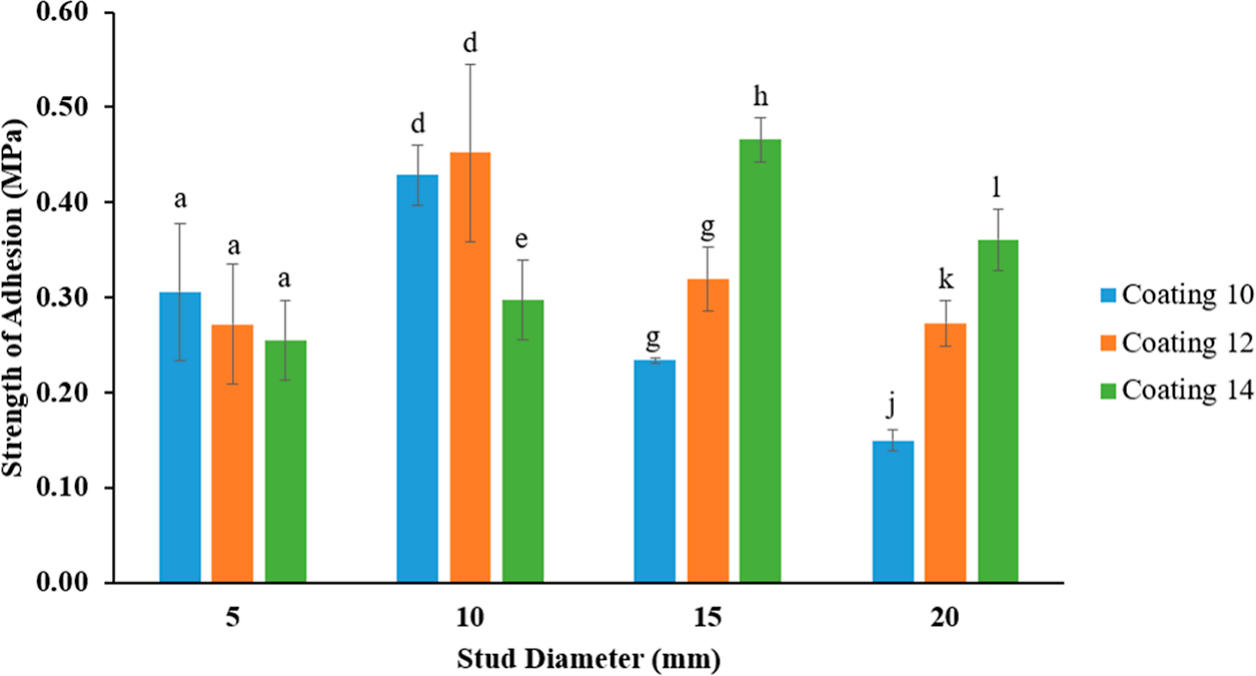
Adhesion strengths of different-sized pseudobarnacles on different coatings after an hour drying.

Figure 6 shows the comparison of adhesion strengths on different coatings and pseudobarnacle diameters. These graphs are not with a constant increase or decrease even though removal strength increases with increasing diameter. For the 5 mm studs, there is no significant difference (*p* > 0.05) between the adhesion strengths of the coatings 10, 12, and 14. While there is no significant difference (*p* > 0.05) between the adhesion strengths of 10 mm studs on coatings 10 and 12, there is a remarkable difference (*p* < 0.05) between the coating 14. 15 mm studs also show no statistical difference (*p* > 0.05) between the coatings 10 and 12, but there is a difference (*p* < 0.05) with the coating 14. All 3 coatings with 20 mm studs differ significantly (*p* < 0.05) from each other.

## 4 Discussion

According to scientific literature, very different barnacle adhesion strengths have been reported so far.^31−33^ In this paper, higher adhesion strengths were obtained using rosin-based coatings, which means that rosin-based coatings did not perform as good as fouling release coatings. The reasons behind these variances are barnacle species, size, age, seasons, and physical conditions of the seawater and test surfaces. Barnacle adhesion strength is strictly based on the antifouling coating types. In short, antifouling paints can be classified into two categories: self-polishing and non-stick technology. In self-polishing antifouling coatings, the binder is soluble in seawater. This means that the coating surface leaches out over time and releases its content. When biocide release is stopped in self-polishing coatings, biofouling organisms adhere and must be cleaned from the surfaces. Otherwise, they cause serious issues such as speed reduction, loss of maneuverability, increased fuel consumption, and increased carbon emission on ships. There are different methods for cleaning biofouling from surfaces. However, most of these techniques use brushes or pressurized water, which can damage the antifouling coatings and surface substrate and reduce its lifetime. The water-jet method is one of them, and the surfaces are cleaned with the high-pressure water.^34^ However, the pressure to be applied should be adjusted so as not to damage the ship hull. On the other hand, in non-stick coatings, the binder or so-called composite is not soluble in seawater. Therefore, the surface characteristics of both coatings are quite different. Nowadays, most effective, and mostly studied non-toxic fouling release surfaces are silicone-based coatings. Due to their low surface energies, poly(dimethyl siloxane) (PDMS)-based coatings are widely used today.^35^ However, PDMS-based coatings have low mechanical properties, and this results in short lifetime. This deficiency is compensated by adding support material, poly(urethane) (PU) and/epoxy segments.^36,37^ In this study, it was aimed to gain similar properties to rosin-based coatings. Therefore, in our study, we examined the adhesion strength of *A. amphitrite* that adhered to self-polishing coatings that do not contain biocide. The results we obtained are especially important in the adjustment of the pressure to be applied in the process of cleaning fouling organisms from the surfaces. Moreover, this study is the first to demonstrate the adhesion strength of *A. amphitrite* on self-polishing coatings.

Dry coating thickness is a parameter that affects the adhesion strength of barnacles.^38^ The dry coating thickness of the prepared coatings was varied between 100 and 178 μm. Wendt et al.^38^ also studied the adhesion strengths of *A. amphitrite* and obtained lower adhesion strengths. However, their study specified on the effects of coating thickness on adhesion strength, and they used silicone-based coating. Although the same species were used in the present study and the study of Wendt et al.,^38^ different results were obtained due to coating thickness and surface characteristics.

Although the supportive materials were added to the coatings prepared with rosin, it was observed that some of the coatings were removed from the metal surface after the field test. In addition to antifouling properties, mechanical properties are also important in coatings. Combining fouling release feature of PDMS with the high mechanical properties of polythiourethane (PTU) resulted in high antifouling and mechanical performance.^39^ Thus, the barnacle and pseudobarnacle adhesion strengths, which were high before, were improved (0.4 to 0.1 MPa and 4.0 to 0.1 MPA, respectively). This means that no matter how high the antifouling property of a substance is, if its physical properties are not sufficient, its lifetime will be short, and it will lose its antifouling property early. Therefore, rosin-based coatings should be modified with components with high mechanical properties to balance antifouling performance and mechanical properties, which will increase the lifetime of the coatings.^35,40^

Apart from the barnacle species and surface properties, adhesive type also affects the pseudobarnacle tests. Barnacles in nature use cement proteins to adhere on to surfaces.^41^ However, in pseudobarnacle tests, commercial adhesives are used to glue aluminum or wooden studs on surfaces. Due to the different chemical structures of these adhesives, it is sometimes not possible to obtain similar results in adhesion tests. For this reason, methodologies that can mimic barnacles specific to the adhesive type should be developed. Besides that, the size and age of the barnacles also affect the adhesion strengths.^21^ Therefore, the diameter of the pseudobarnacle used should be specified in the methodology.

When barnacle and pseudobarnacle adhesion strength results were compared, it was found that the coatings 10 and 12, in which a 10 mm diameter pseudobarnacle was applied and dried for an hour, gave a similar adhesion strength with 10 mm diameter live barnacles. In the tests carried out on the live barnacles after the field test, the 10 mm diameter barnacles attached to the coating 10 were detached from the surface with 34 N and the strength of adhesion was calculated as 0.43 MPa, while the 36 N was measured and 0.46 MPa was calculated on the coating 12. Similarly, in the pseudobarnacle tests performed, 34 N was measured and 0.43 MPa was calculated for the coating 10, while 35 N was measured for the coating 12 and 0.45 MPa was calculated. The methodology followed is explained in Figure S11.

Other than that, correlation of diameters and removal strengths between all barnacle and pseudobarnacle adhesion tests on coating 10, 12, and 14 gave matching results (Table 9). Paint manufacturers can use these data before starting their trials. However, it should not be forgotten that these values vary with different physicochemical conditions of seawater.

**Table 9:** Correlations of Diameters and Removal Strengths between Barnacles and Pseudobarnacles

coating no.	correlation diameters between barnacles vs pseudobarnacles	correlation removal strength between barnacles vs pseudobarnacles
10	0.845	0.625
12	0.943	0.799
14	0.950	0.663

## 5 Conclusions

In this study, biocide-free self-polishing coatings were prepared, and field tests were performed on these coatings. Because of these field tests, the adhesion strengths of *A. amphitrite* attached to the surfaces were examined. Furthermore, pseudobarnacle adhesion tests were performed to mimic barnacle adhesion strengths in cases where field tests could not be performed. According to the tests conducted on the coating 12 [40% (w/w) rosin, 40% (w/w) xylene, 20% (w/w) BaSO_4_], the barnacle adhesion strength was found to be 0.46 MPa, while pseudobarnacle adhesion strength was found to be 0.45 MPa. When all the adhesion strength tests results were compared, similar correlations of diameter and removal strengths between barnacles and pseudobarnacles were obtained.

In conclusion, the development of eco-friendly antifouling coatings is a challenging process due to many factors such as environmental conditions, physicochemical parameters of seawater, and testing organisms. Therefore, pseudobarnacle adhesion tests for the development of eco-friendly antifouling coatings are of great importance. However, standardized conditions should be carefully studied to prevent possible differences among laboratories. Moreover, based on field tests, a pseudobarnacle adhesion methodology was developed to mimic barnacles and the correlation between barnacle and pseudobarnacle tests was examined.

## Supporting Information Available

Coating formulations; images of field test results; and additional methodology for pseudobarnacle adhesion strength test (PDF)
