# Supplementary material for: First Barnacle (Amphibalanus amphitrite) Adhesion Strength Data on the Self-Polishing Coatings Off the Aegean Sea
Source: ACS Omega. 2023 Sep 8;8(37):33675–83. doi: 10.1021/acsomega.3c03948 (PMC10515341; doi:10.1021/acsomega.3c03948)
Supplement: Supplementary file 1 — ao3c03948_si_001.pdf [file ao3c03948_si_001.pdf]

Supporting Information

First Barnacle (*Amphibalanus amphitrite*) Adhesion Strength Data on the Self-Polishing Coatings off the Aegean Sea

Ibrahim Kirkiz[1] *- Levent Cavas*[1,2*]

[1]Dokuz Eylül University, Graduate School of Natural and Applied Sciences, Department of Biotechnology, Kaynaklar Campus, 35390, İzmir, Türkiye.

[2]Dokuz Eylül University, Faculty of Science, Department of Chemistry (Biochemistry Division), Kaynaklar Campus, 35390, İzmir, Türkiye.

Table S1. Prepared coating and their contents.

Coating	Materials (w/w %)							
	**Rosin**	**Xylene**	**Zinc rosinate**	**PVB**	**Distilled Water**	**$CaCO_3$**	**ZnO**	**$BaSO_4$**
1	25	75	-	-	-	-	-	-
2	50	50	-	-	-	-	-	-
3	75	25	-	-	-	-	-	-
4	-	75	25	-	-	-	-	-
5	-	50	50	-	-	-	-	-
6	-	25	75	-	-	-	-	-
7	-	-	-	36	64	-	-	-
8	-	-	-	18	72	-	-	-
9	-	-	-	9	81	-	-	-
10	40	40	-	-	-	20	-	-
11	40	40	-	-	-	-	20	-
12	40	40	-	-	-	-	-	20
13	40	40	-	-	-	10	10	-
14	40	40	-	-	-	10	-	10
15	40	40	-	-	-	-	10	10
16	-	40	40	-	-	20	-	-
17	-	40	40	-	-	-	20	-
18	-	40	40	-	-	-	-	20
19	-	40	40	-	-	10	10	-
20	-	40	40	-	-	10	-	10
21	-	40	40	-	-	-	10	10
22	35	35	-	-	-	10	10	10
23	-	35	35	-	-	10	10	10

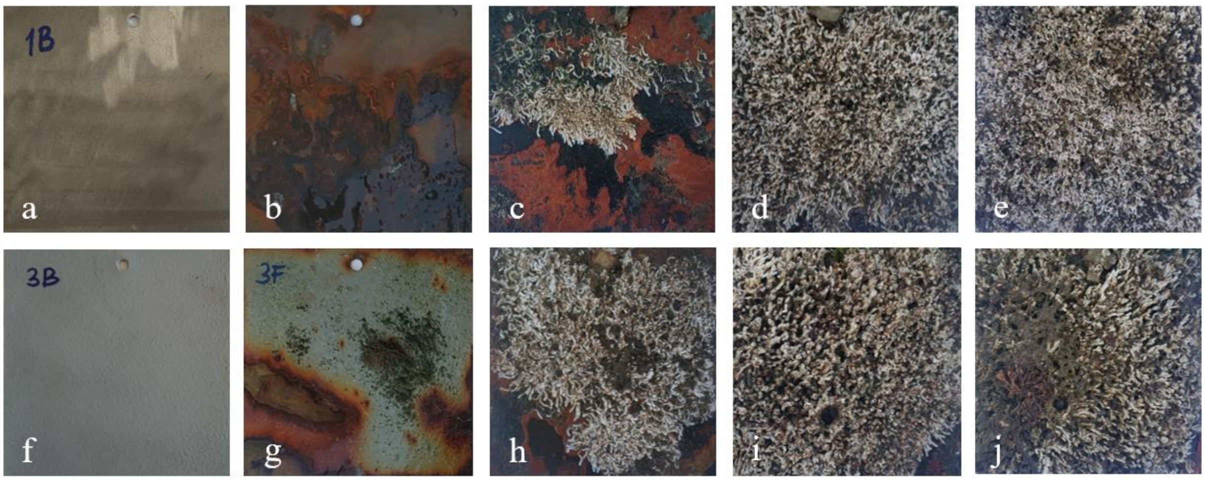

Figure S1. Field test results of uncoated plate (a) before immersion, (b) after 2 weeks, (c) after 4 weeks, (d) after 7 weeks, (e) after 10 weeks; anti-corrosive primer coated plate (f) before immersion, (g) after 2 weeks, (h) after 4 weeks, (i) after 7 weeks, (j) after 10 weeks.

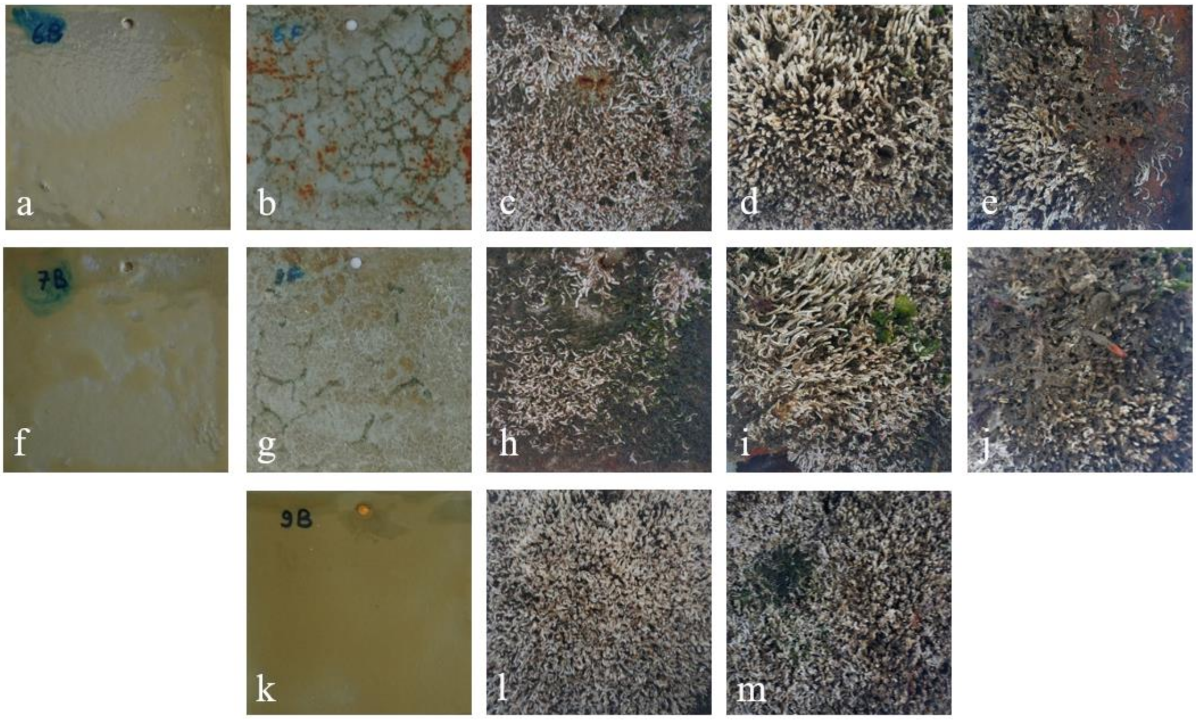

Figure S2. Field test results of Coating-1 (a) before immersion, (b) after 2 weeks, (c) after 4 weeks, (d) after 7 weeks, (e) after 10 weeks; Coating-2 (f) before immersion, (g) after 2 weeks, (h) after 4 weeks, (i) after 7 weeks, (j) after 10 weeks; Coating-3 (k) before immersion, (l) after 3 weeks, (m) after 6 weeks.

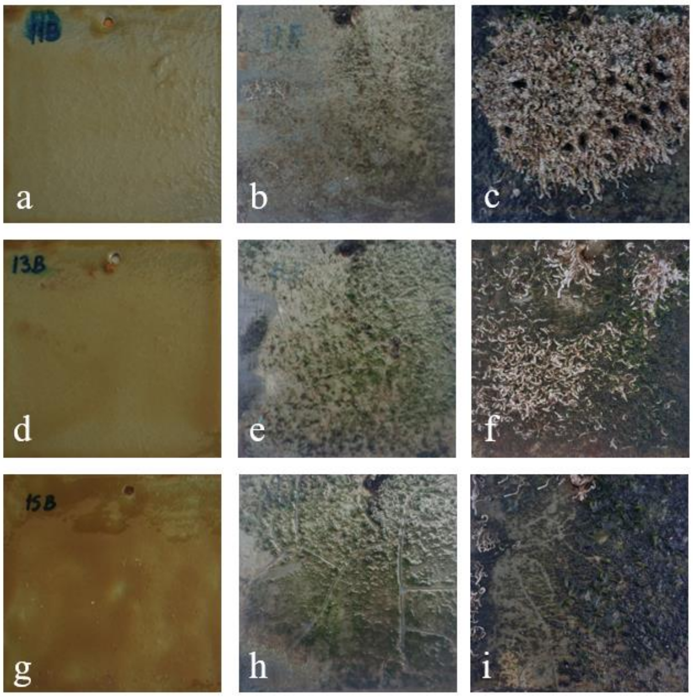

Figure S3. Field test results of Coating-4 (a) before immersion, (b) after 3 weeks, (c) after 6 weeks; Coating-5 (d) before immersion, (e) after 3 weeks, (f) after 6 weeks; Coating-6 (g) before immersion, (h) after 3 weeks, (i) after 6 weeks.

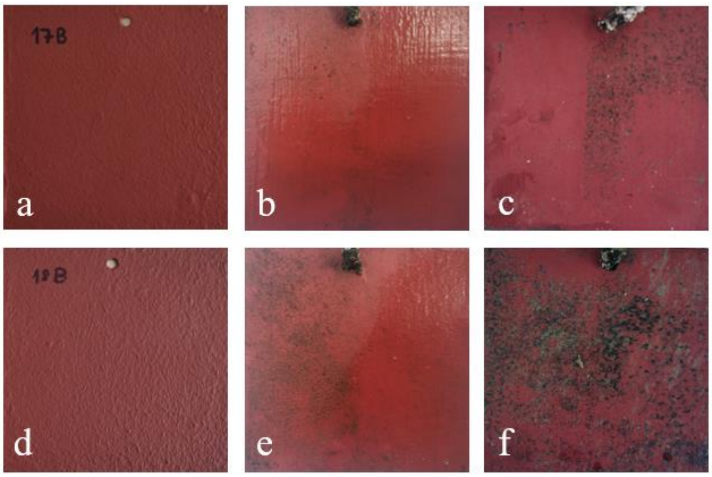

Figure S4. Field test results of commercial paint (a, d) before immersion, (b, e) after 3 weeks, (c, f) after 6 weeks.

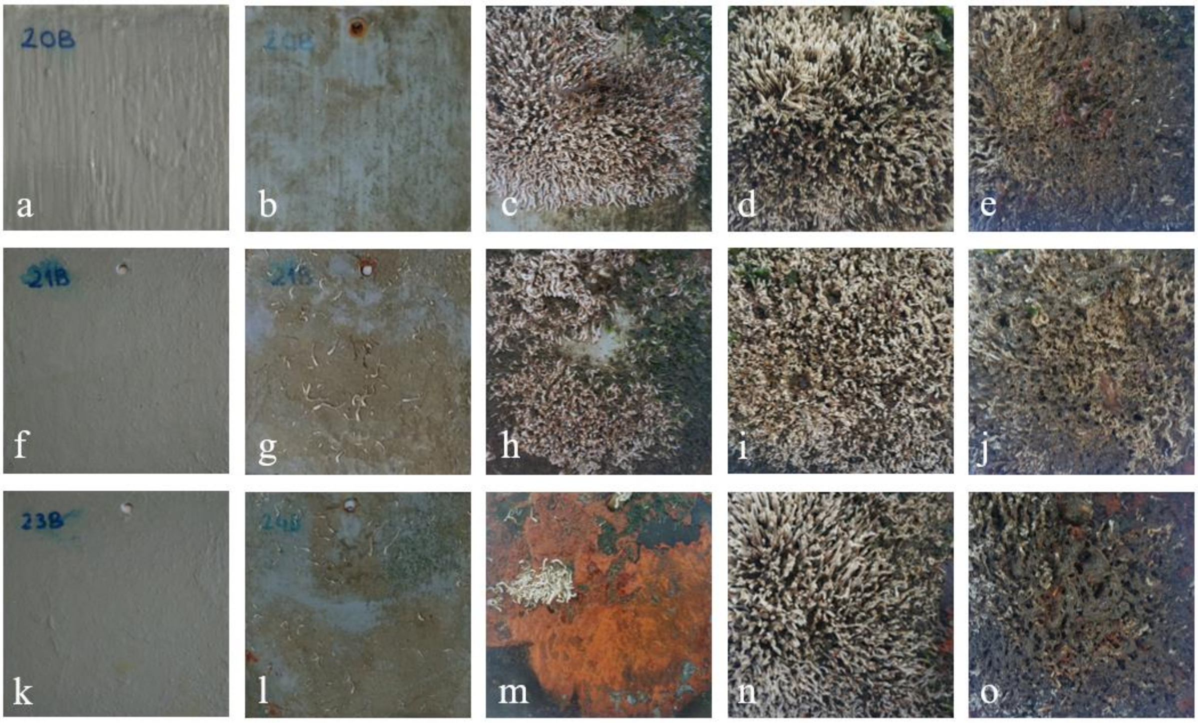

Figure S5. Field test results of Coating-7 (a) before immersion, (b) after 2 weeks, (c) after 4 weeks, (d) after 7 weeks, (e) after 10 weeks; Coating-8 (f) before immersion, (g) after 2 weeks, (h) after 4 weeks, (i) after 7 weeks, (j) after 10 weeks; Coating-9 (k) before immersion, (l) after 2 weeks, (m) after 4 weeks, (n) after 7 weeks, (o) after 10 weeks.

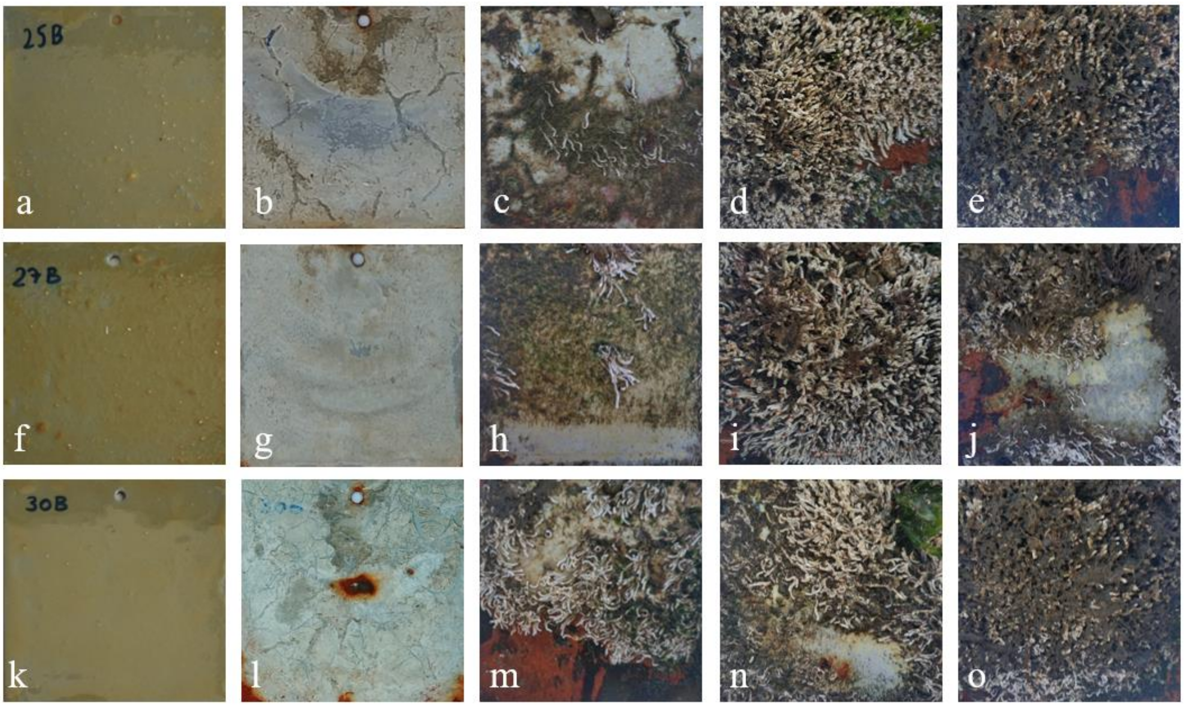

Figure S6. Field test results of Coating-10 (a) before immersion, (b) after 2 weeks, (c) after 4 weeks, (d) after 7 weeks, (e) after 10 weeks; Coating-11 (f) before immersion, (g) after 2 weeks, (h) after 4 weeks, (i) after 7 weeks, (j) after 10 weeks; Coating-12 (k) before immersion, (l) after 2 weeks, (m) after 4 weeks, (n) after 7 weeks, (o) after 10 weeks.

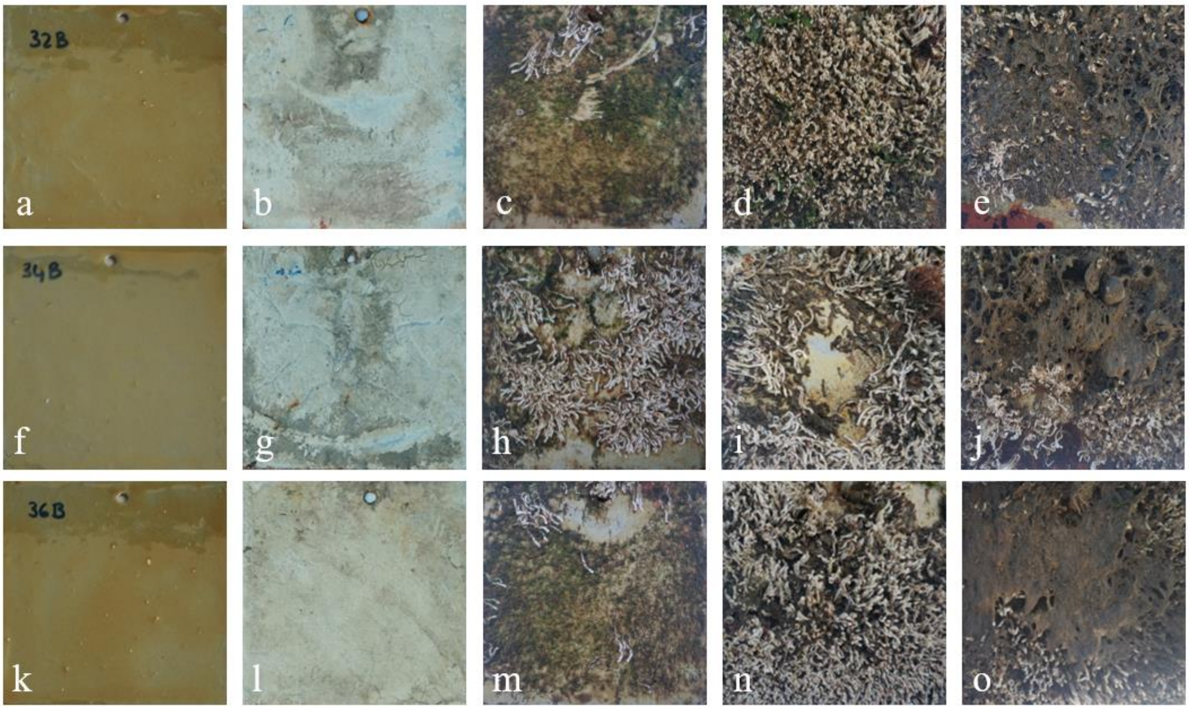

Figure S7. Field test results of Coating-13 (a) before immersion, (b) after 2 weeks, (c) after 4 weeks, (d) after 7 weeks, (e) after 10 weeks; Coating-14 (f) before immersion, (g) after 2 weeks, (h) after 4 weeks, (i) after 7 weeks, (j) after 10 weeks; Coating-15 (k) before immersion, (l) after 2 weeks, (m) after 4 weeks, (n) after 7 weeks, (o) after 10 weeks.

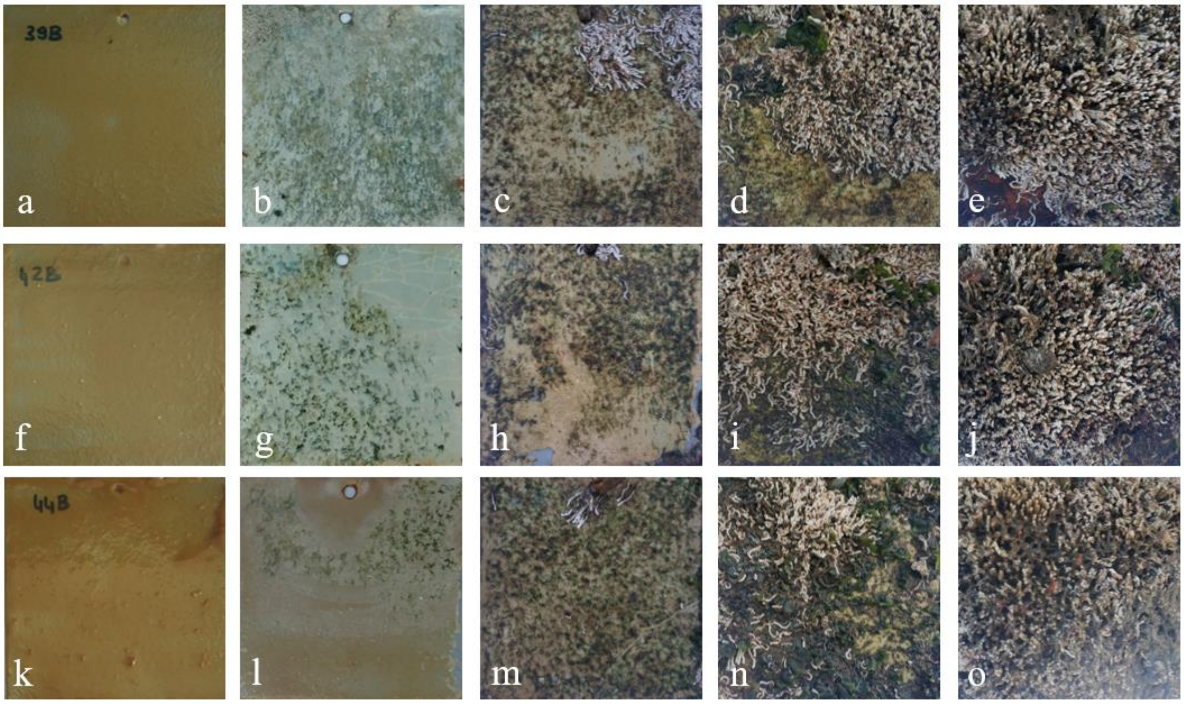

Figure S8. Field test results of Coating-16 (a) before immersion, (b) after 2 weeks, (c) after 4 weeks, (d) after 7 weeks, (e) after 10 weeks; Coating-17 (f) before immersion, (g) after 2 weeks, (h) after 4 weeks, (i) after 7 weeks, (j) after 10 weeks; Coating-18 (k) before immersion, (l) after 2 weeks, (m) after 4 weeks, (n) after 7 weeks, (o) after 10 weeks.

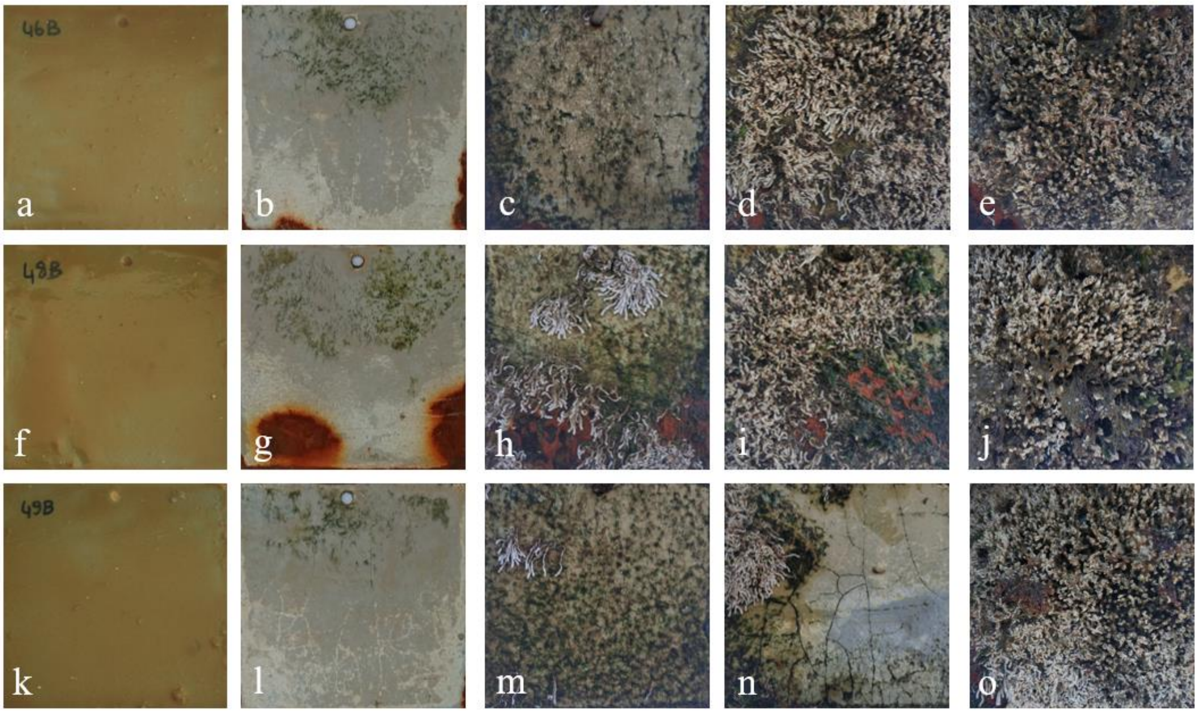

Figure S9. Field test results of Coating-19 (a) before immersion, (b) after 2 weeks, (c) after 4 weeks, (d) after 7 weeks, (e) after 10 weeks; Coating-20 (f) before immersion, (g) after 2 weeks, (h) after 4 weeks, (i) after 7 weeks, (j) after 10 weeks; Coating-21 (k) before immersion, (l) after 2 weeks, (m) after 4 weeks, (n) after 7 weeks, (o) after 10 weeks.

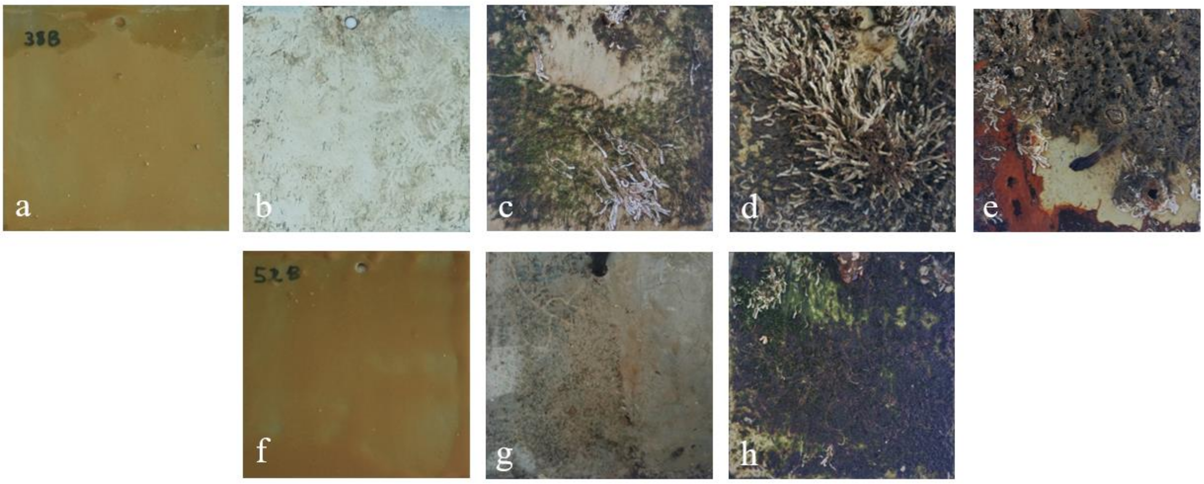

Figure S10. Field test results of Coating-22 (a) before immersion, (b) after 2 weeks, (c) after 4 weeks, (d) after 7 weeks, (e) after 10 weeks; Coating-23 (f) before immersion, (g) after 3 weeks, (h) after 6 weeks.

1. Metal plates with the size of 10x10 cm^2 were obtained. These plates were first sanded and then, the surface was cleaned of dust.
2. Then the entire surface was coated with anti-corrosive primer.
3. After the primer was completely dried, coating 12 was prepared and applied to the plates. Coated plates were left to dry in the open for about 2 weeks.
4. After the coatings fully dried, pseudobarnacles were prepared from wood with a diameter and height of 10 mm. Then, the surfaces of the pseudobarnacles were sanded to obtain a smooth and flat surface.
5. A two-component epoxy was mixed by taking equal amounts of its components. Then, this mixture was applied as a thin film on a surface of the pseudobarnacles and adhered to the coating surface without waiting.
6. Excess epoxy was removed without damaging the coating with the help of a cotton swab and acetone. Then, the epoxy was left to dry for about an hour.
7. Before starting the pseudobarnacle adhesion test, 8-10 mm rod and appropriate probe were mounted on the dynamometer (Loyka NK 200).
8. The plate and the dynamometer were brought to a parallel position and a constant force was applied to the pseudobarnacle with the dynamometer.
9. The force at which the pseudobarnacles were completely detached from the surface was recorded and used in calculations to calculate adhesion strength.

Figure S11. The methodology used in the pseudobarnacle adhesion tests.
